# Intranasal acupuncture for allergic rhinitis: A systematic review and meta-analysis

**DOI:** 10.1097/MD.0000000000040305

**Published:** 2024-11-08

**Authors:** Yongjun Li, Yijie Wang, Yuan Liang, Xiuying Si, Zhixiang Li, Youpeng Wang

**Affiliations:** a Shanxi Traditional Chinese Medical Hospital, Taiyuan, China; b The Second Affiliated Hospital of Heilongjiang University of Chinese Medicine, Harbin, China; c Qingdao Hiser Hospital Affiliated of Qingdao University (Qingdao Traditional Chinese Medicine Hospital), Qingdao, China; d Heilongjiang University of Chinese Medicine, Harbin, China.

**Keywords:** allergic rhinitis, intranasal acupuncture, meta-analysis

## Abstract

**Background:**

To evaluate the efficacy of intranasal acupuncture as a treatment for allergic rhinitis (AR) through a comprehensive review.

**Methods:**

Comprehensive searches were performed in both Chinese (CNKI, VIP, CBM, and Wanfang) and English databases (PubMed, Embase, Cochrane Library, and Web of Science) to gather randomized controlled trials available from the inception of the database until August 2024. The primary outcomes considered were the effectiveness rate, visual analog scale score, total nasal symptom scores, total nonnasal symptom scores, Rhinoconjunctivitis Quality-of-Life Questionnaire score, adverse effects, and follow-up observations. The quality of each study was assessed using the Cochrane Collaboration risk of bias tool, and data analysis was conducted using RevMan 5.4 software.

**Results:**

This study incorporated 14 articles involving a total of 1009 patients. The meta-analysis revealed that patients with AR who underwent intranasal acupuncture experienced more significant improvements compared to the control group. Notably, the treatment considerably improved both nasal and nonnasal symptoms, along with the patients’ quality of life. Moreover, during the follow-up, it was noted that intranasal acupuncture patients had a lower recurrence rate compared to the control group, indicating better long-term effects in alleviating symptoms like nasal congestion, runny nose, and sneezing. Nonetheless, there was no marked improvement of nasal itching. It’s noteworthy that some adverse effects were reported, but all were mild.

**Conclusions:**

The findings suggest that intranasal acupuncture serves as an effective intervention for AR, particularly in alleviating both nasal and nonnasal symptoms and enhancing quality of life. However, these positive outcomes should be approached with caution, and further high-quality and extensive studies to substantiate these results are warranted.

## 1. Introduction

Allergic rhinitis (AR), characterized as a noninfectious inflammatory ailment affecting the nasal mucosa, is triggered by immunoglobulin E (IgE) and a variety of immunocompetent cells and factors upon exposure to allergens in susceptible individuals.^[1]^ This condition can induce autonomic nervous system abnormalities, manifesting in nasal mucosal hyperemia and increased gland secretions. The primary clinical symptoms encompass intermittent or persistent nasal congestion, nasal itching, sneezing, and a runny nose,^[2]^ which can markedly deteriorate patients’ quality of life.^[3]^ Currently, AR is prevalent in 10% to 20% of the global population, establishing itself as a significant chronic nasal malady.^[4]^ Given its notable surge in recent years, AR has evolved into a common chronic respiratory inflammatory condition,^[5]^ imposing a substantial economic and social burden.^[6]^

To prevent and manage AR, strategies include minimizing exposure to allergens and irritants, utilizing medications, and immunotherapy treatment.^[7]^ Pharmacological interventions remain the preferred treatment approach, utilizing glucocorticoids, antihistamines, and antileukotrienes as staple medications in AR management.^[8]^ However, these pharmaceuticals, though effective in alleviating rhinitis symptoms swiftly, do not offer a cure for AR. Furthermore, patients often exhibit reluctance towards these treatments due to the significant side effects and high recurrence rates associated with prolonged use.^[9]^ Consequently, a growing number of researchers are exploring alternative treatments in the realm of complementary and alternative medicine therapies.^[9–11]^

Acupuncture, a pivotal component of Traditional Chinese Medicine, is a prime example of a longstanding complementary and alternative medicine therapy. In treating AR, acupuncture principally moderates serum IgE levels, harmonizes the Th1/Th2 equilibrium, diminishes eosinophil infiltration, and curtails the release of inflammatory mediators, thereby balancing nasal mucosal neuropeptides.^[12]^ This therapy demonstrates anti-inflammatory properties, alleviating nasal mucosal inflammation and other symptoms through the effects on immune, neurological, and inflammatory responses.^[13]^ Given the escalating interest in acupuncture, numerous studies have focused on specialized acupuncture points to augment its efficacy,^[14]^ indicating that intranasal acupuncture holds potential merits in AR treatment.^[15]^ However, the scarcity of evidence-based research hinders a comprehensive evaluation of its effectiveness in addressing AR.

**Figure 1.**
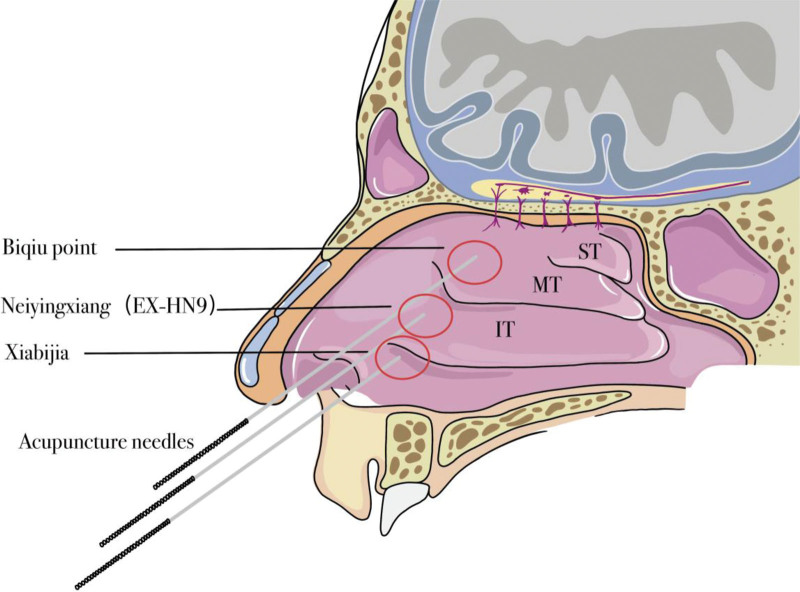
The anatomical location of Neiyingxiang (EX-HN9), Biqiu, and Xiabijia.

Intranasal acupuncture is a unique approach to treating AR, targeting specific points such as Neiyingxiang (EX-HN9), Biqiu, and Xiabijia, whose anatomical locations are depicted in Figure 1. Recently, a surge in studies have vouched for the efficacy and safety of conventional acupuncture points in managing AR.^[16]^ Following a thorough analysis of Traditional Chinese Medicine theories and the characteristic pathogenesis of AR, Chinese acupuncture specialists have formulated an innovative treatment strategy. This approach seeks to enhance clinical outcomes by employing specific acupuncture points within the nasal cavity for AR treatment.^[17]^ Several randomized controlled trials (RCTs) have explored this method, affirming its precise clinical impact.^[18]^ Nevertheless, a void exists in systematic reviews in the clinical arena. Therefore, this review endeavors to shed light on the safety and effectiveness of intranasal acupuncture in AR treatment, aiming to furnish patients who experience adverse reactions to medications or those opting for acupuncture therapy with superior quality acupuncture alternatives.

## 2. Materials and methods

### 2.1. Ethics statement

This study is a literature review, not human or animal experiments, so it does not require ethical approval from the ethics committee. This review was conducted in accordance with the guidelines outlined in the Cochrane Handbook for Systematic Reviews of Interventions and adheres strictly to the criteria stipulated in the Preferred Reporting Items for Systematic Reviews and Meta-Analyses (PRISMA) 2020 statement, an enhanced guideline for articulating systematic reviews. The protocol has been duly registered with PROSPERO, bearing the registration number CRD42023407716 (see Appendix 1, Supplemental digital content, http://links.lww.com/MD/N869, which shows the PROSPERO register).

### 2.2. Search strategy

A comprehensive search was performed covering scientific databases such as PubMed, Embase, Web of Science, Cochrane Library, CNKI, Wanfang, VIP, and CBM to identify RCTs studying the effects of intranasal acupuncture on AR, from the time of their inception until August 2024, without any language restrictions. The search strategy employed the following terms: (“Rhinitis, Allergic”[Mesh] OR “Seasonal Allergic Rhinitis”[Mesh] OR “Rhinitis, Allergic, Perennial”[Mesh]) AND (“Intranasal Acupuncture”[Mesh]). An in-depth overview of the search strategy can be found in supplementary digital content (see Appendix 2, Supplemental digital content, http://links.lww.com/MD/N869, which illustrates the literature search strategy). Additionally, the reference lists of the retrieved studies were scrutinized to pinpoint other potentially eligible trials for inclusion.

### 2.3. Inclusion and exclusion criteria

To align with the objectives of this study, we defined the inclusion criteria as follows: population—participants must satisfy the diagnostic benchmarks for AR^[19]^; intervention—the primary therapeutic procedure should be intranasal acupuncture; design—RCTs only; data—must contain complete original data.

The exclusion criteria were established as follows: submissions in the form of conference abstracts or systematic reviews; duplicated publications; studies involving animal trials or nonclinical experiments; articles presenting problematic experimental designs; unavailable full texts.

### 2.4. Study selection and data extraction

A pair of investigators undertook the task of independently scouring databases and evaluating and verifying the articles selected for inclusion. Any disputes were resolved by consulting a third investigator, who reviewed the content and facilitated discussion to arrive at a conclusive judgement. Data extraction encompassed details such as the first author’s name, publication year, country of origin, case distribution across groups, demographic details (age, gender), the course of the study, interventions deployed, and duration of treatment and follow-up.

### 2.5. Assessment of risk of bias

Two separate reviewers utilized the Cochrane Collaboration’s risk of bias tool to independently assess the risk of bias in each RCT. This assessment encompassed several facets such as random sequence generation, allocation concealment, participant and personnel blinding, outcome assessment blinding, incomplete outcome data, selective reporting, and other potential biases. These elements were categorized as either “low risk,” “high risk,” or “unclear risk.” A third reviewer mediated and resolved any discrepancies in opinion.

### 2.6. Statistical analysis

The meta-analysis was conducted using Rev Man 5.4 software provided by the Cochrane Network. Pooled numerical data were articulated as relative risk (RR) with a 95% confidence interval (CI), whereas pooled continuous variables were conveyed as mean standard deviation (MD) with a 95%CI. A *P*-value less than .05 was considered the threshold for statistical significance. The Q test (determined through *P*-value) and the I-square test (I2) were used to gauge the extent of heterogeneity. In cases where the tests signified insignificant heterogeneity (*P* ≥ .1, I2 < 50%), a fixed-effects model was applied for analysis. Conversely, when substantial statistical heterogeneity was noted between studies (*P* < .1, I2 > 50%), a random effects model was implemented, followed by a meta-analysis. If more than 10 studies were incorporated, a funnel plot was employed to detect any publication bias.

## 3. Results

### 3.1. Study selection

**Figure 2.**
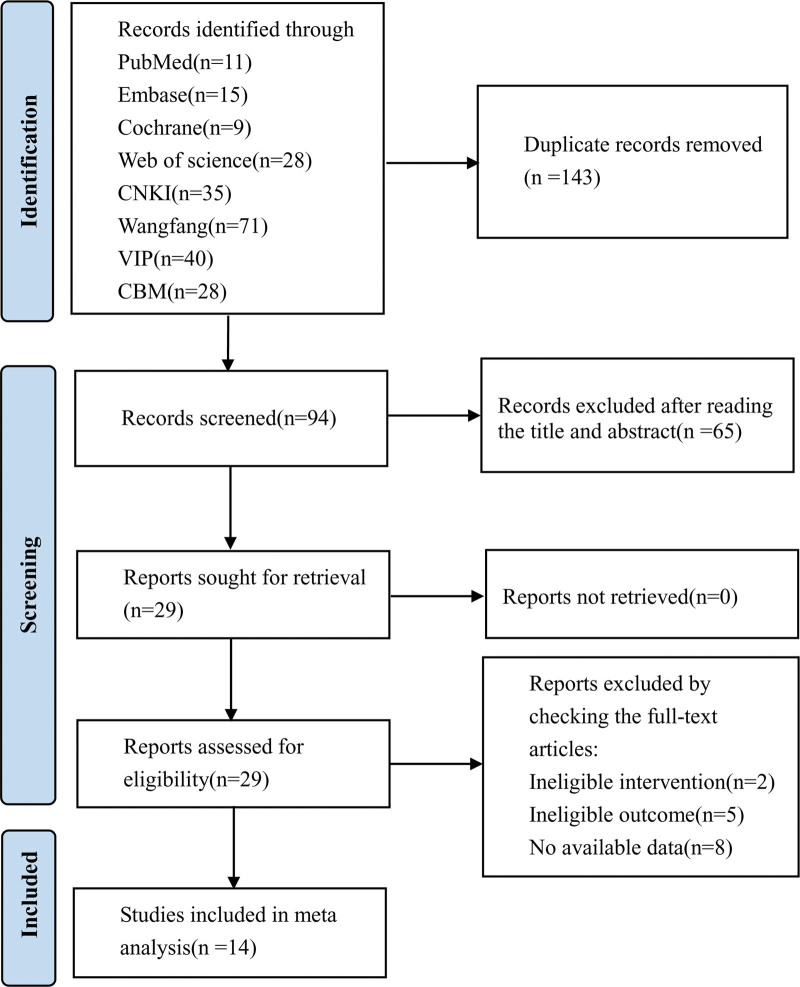
The results of literature screening and the process.

Initially, 237 articles were identified. After eliminating duplicate publications, a total of 143 studies were excluded. Through reviewing titles and abstracts, the citation pool was narrowed down to 29 promising studies. From these, 15 articles were subsequently discarded due to inadequate interventions, outcomes, or unavailable data. Consequently, 14 trials were selected for this analysis,^[20–33]^ involving 1009 patients. A graphical representation of the literature search process is illustrated in Figure 2.

### 3.2. Study characteristics

This analysis incorporates 14 articles, all of which are RCTs conducted in China. Notably, only 1 article is published in English,^[20]^ with the remaining available in Chinese. The publication period spans from 2005, represented by Liu Qiaoping’s study, to 2022, which includes the rest of the trials. The analysis covers 1009 participants, aged 18 to 65, 521 in the intranasal acupuncture group and 488 in the control group. The disease durations range from 3 months to 20 years, and treatment periods extend from 2 weeks to 2 months. Treatment frequencies in the experimental group varied, primarily occurring daily or bi-daily, utilizing intranasal acupuncture exclusively or in conjunction with control group measures. The control group predominantly utilized nasal sprays as dictated by relevant guidelines, with variations including extra-nasal acupuncture, acupoint autohemotherapy, and Traditional Chinese Medicine decoctions, among others. Follow-up periods ranged between 1 and 6 months across 7 studies. Comprehensive details of the studies are presented in Table 1.

**Table 1:** Basic information of the included literature.

Year	Author	Study types	Country	Cases		Ages(mean ± sd)		Gender (Male/female)		Course		Intervention		Period of treatment	Follow-up time(months)
				Treatment group	Control group	Treatment group(year)	Control group(year)	Treatment group	Control group	Treatment group	Control group	Treatment group	Control group		
2022	Li-Li Liu^[20]^	RCT	China	60	30	40.98 ± 10.35	41.46 ± 10.99	31/29	15/15	8.48 ± 3.20 years	7.26 ± 3.01 years	Intranasal acupuncture at the Neiyingxiang and Biqiu point	Budesonide nasal spray and loratadine	2 weeks	1
2022	Liu Jin^[21]^	RCT	China	36	36	42.33 ± 9.72	40.22 ± 8.03	21/15	20/16	11.97 ± 4.93 years	10.56 ± 3.26 years	Nasal spray fluticasone propionate and intranasal acupuncture at the Neiyingxiang and Biqiu point	Nasal spray fluticasone propionate	4 weeks	3
2022	Li Yan^[22]^	RCT	China	35	35	30.33 ± 1.64	29.88 ± 1.03	18/17	19/16	4.73 ± 2.03 years	4.69 ± 1.25 years	Intranasal acupuncture at the Neiyingxiang and Biqiu point	Pressing needle at Yintang and bilateral Yingxiang Bitong	2 weeks	6
2021	Zang Jianghong^[23]^	RCT	China	43	44	40.32 ± 8.50	38.24 ± 8.16	21/22	25/19	6.24 ± 2.06 years	5.59 ± 2.13 years	Intranasal acupuncture at the Neiyingxiang and Biqiu point	Acupoint autohemotherapy	2 months	6
2022	Tao Jinghua^[24]^	RCT	China	30	30	34.47 ± 10.57	34.37 ± 11.58	13/17	14/16	5.70 ± 2.47 years	5.97 ± 1.85 years	Intranasal acupuncture at the Neiyingxiang point	Acupuncture at the Yingxiang point	2 weeks	–
2021	Li Wentao^[25]^	RCT	China	30	30	38.63 ± 9.03	37.87 ± 9.07	16/14	15/15	11.03 ± 2.93 years	11.57 ± 2.57 years	Intranasal acupuncture at the Neiyingxiang and Biqiu point	Loratadine	2 weeks	–
2021	Li Yan^[26]^	RCT	China	33	33	38 ± 4	36 ± 4	17/16	17/16	11.13 ± 2.61 years	10.74 ± 2.50 years	Intranasal acupuncture at the Neiyingxiang point	Acupuncture at the Yingxiang point	2 weeks	4
2020	Gong Zheng^[27]^	RCT	China	78	80	37.99 ± 9.62	38.28 ± 11.29	36/45	33/47	6.16 ± 3.86 years	6.21 ± 3.99 years	Intranasal acupuncture at the Neiyingxiang point	Budesonide nasal spray and loratadine	2 weeks	–
2020	Bian Fangzi^[28]^	RCT	China	25	22	39.20 ± 10.51	46.00 ± 11.90	14/11	10/12	–	–	Intranasal acupuncture at the Neiyingxiang point	Acupuncture at the Yingxiang point	2 weeks	1
2019	Li Yan^[29]^	RCT	China	37	38	36.64 ± 2.19	38.35 ± 2.301	20/17	19/19	10.43 ± 1.08 months	10.83 ± 1.05 months	Intranasal acupuncture at the Neiyingxiang and Biqiu point	Loratadine	2 weeks	6
2019	Ren Xuefei^[30]^	RCT	China	30	30	33.83 ± 9.52	36.33 ± 8.88	12/18	14/16	–	–	Traditional Chinese medicine decoction and intranasal acupuncture at the Neiyingxiang, Biqiu and Xiabijia point	Traditional Chinese medicine decoction	2 weeks	–
2018	Gong Zheng^[31]^	RCT	China	24	20	39.58 ± 10.98	29.75 ± 9.46	8/16	7/13	21.33 ± 12.17 months	24.11 ± 10.97 months	Intranasal acupuncture at the Neiyingxiang point	Budesonide nasal spray and loratadine	2 weeks	–
2018	Yuan Haizhou^[32]^	RCT	China	30	30	34.1 ± 6.8	33.8 ± 6.2	11/19	10/20	4.7 ± 2.2 years	4.8 ± 2.0 years	Intranasal acupuncture at the Neiyingxiang and Biqiu point	Budesonide nasal spray	20 days	–
2005	Liu Qiaoping^[33]^	RCT	China	30	30	32.63 ± 13.58	35.70 ± 11.43	21/9	18/12	4.41 ± 4.00 years	4.76 ± 4.84 years	Intranasal acupuncture at the Biqiu point	Loratadine	10 days	–

RCT = randomized controlled trial.

### 3.3. Risk and bias

**Figure 3.**
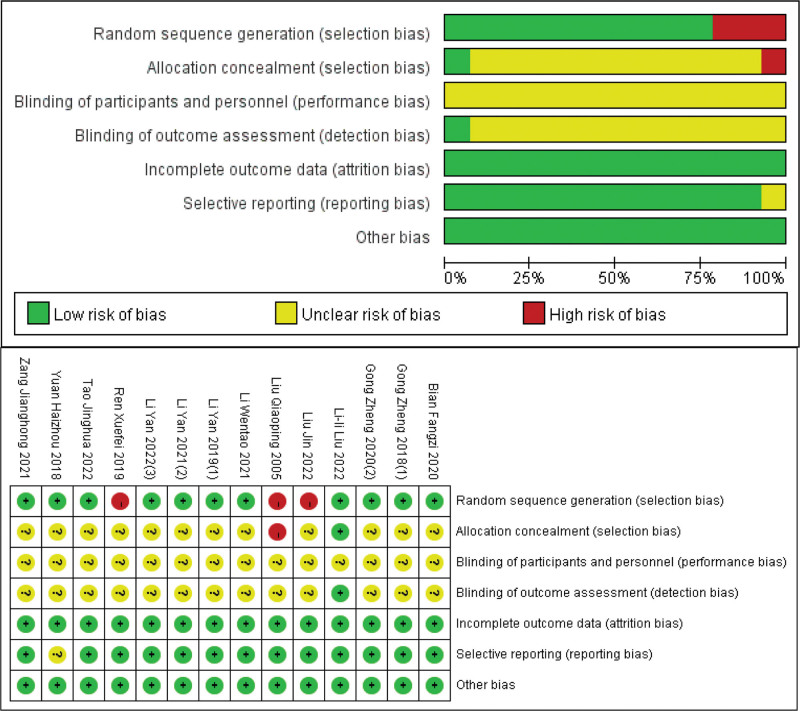
Risk of bias summary and graph.

Figure 3 showcases the risk of bias assessment, as deduced from the Cochrane Collaboration risk of bias tool outcomes. Eleven studies provided details on the randomization process,^[20,22–29,31,32]^ with 1 clearly delineating the allocation concealment process.^[20]^ Blinding of participants and personnel was not feasible given the distinct treatment approaches involved. However, Lili Liu’s trial did implement a method to blind outcome assessments. All studies detailed the reasons for dropout occurrences when relevant. Protocols were followed rigorously in 13 trials, with the remainder not offering significant insights for evaluating publication bias.

### 3.4. Effective rate

**Figure 4.**
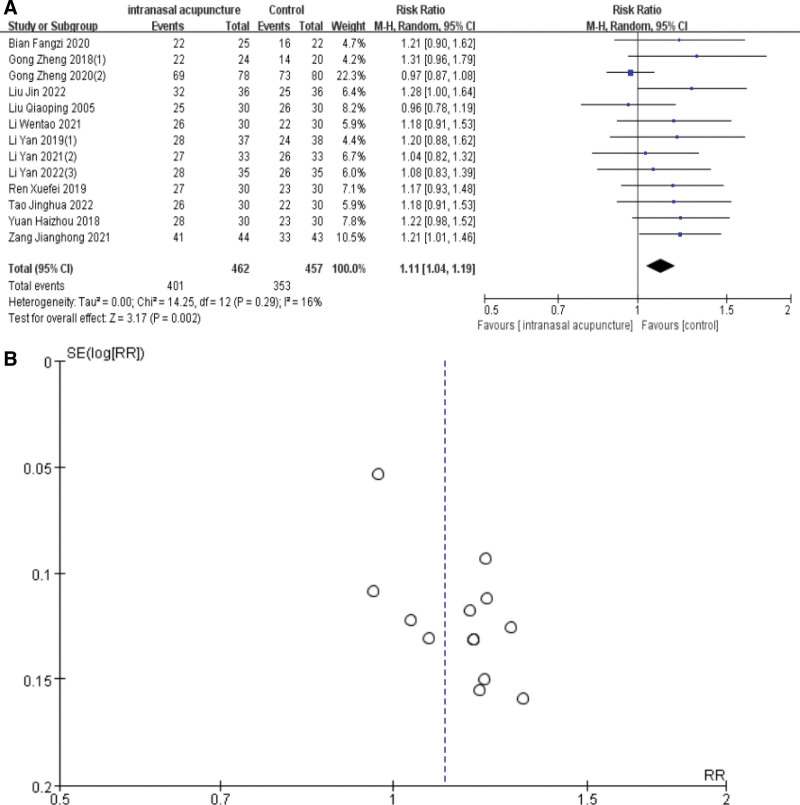
Forest plot and sensitivity analysis of meta-analysis on effective rate.

Barring the study by Li-Li Liu,^[20]^ the remaining 13 trials reported efficacy using dichotomous variables. The overall efficacy rates for the intranasal acupuncture and control groups were 86.8% (401/462) and 77.2% (353/457), respectively. The meta-analysis revealed a significant improvement in the effective rate of AR treatment with intranasal acupuncture, denoted by [RR = 1.11, 95%CI (1.04, 1.19), *P* = .002], and a low heterogeneity among the trials reporting these figures (I2 = 14%, *P* = .29). Furthermore, the risk of publication bias was analyzed using a funnel plot due to the inclusion of more than 10 RCTs, indicating a low publication bias as evidenced by the symmetrical distribution in the plot (Fig. 4).

### 3.5. Visual analog scale (VAS) scores

**Figure 5.**
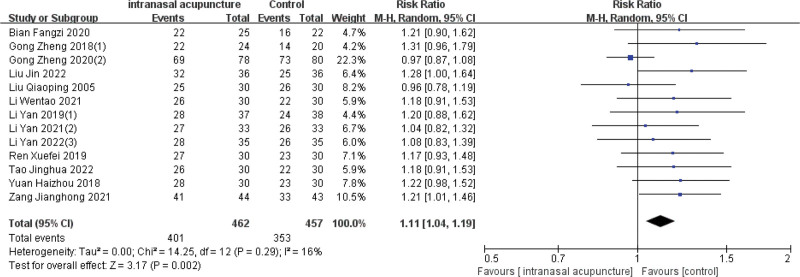
Forest plot of meta-analysis on VAS scores. VAS = visual analog scale.

Five trials discussed the VAS scores, represented as continuous variables within the incorporated articles. Applying the random-effects model (I2 = 88%, *P* < .00001) for pooling the effect size demonstrated a statistically significant difference between the groups [MD = −2.67, 95%CI (−5.08, −0.26), *P* = .03]. This meta-analysis underscores the superior efficacy of intranasal acupuncture in alleviating severe AR compared to the control group (Fig. 5).

### 3.6. Total nasal symptom scores (TNSS)

**Figure 6.**
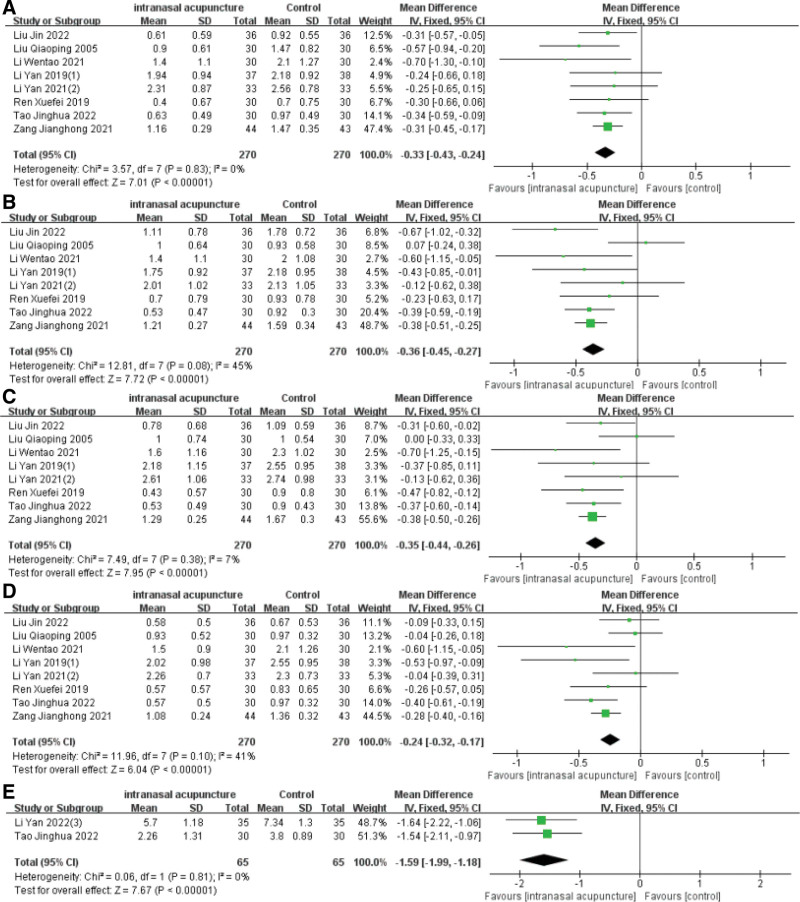
Forest plot of meta-analysis on TNSS. TNSS = total nasal symptom scores.

Eight papers documented individual nasal symptom scores including nasal congestion, rhinorrhea, sneezing, and nasal itching, presented as continuous data. Employing the fixed-effects model to aggregate the effect size highlighted a statistically significant discrepancy between the groups, with varying mean differences and confidence intervals for each symptom, all indicating a *P*-value of less than .0001. The meta-analysis substantiates the marked superiority of intranasal acupuncture over the control group in mitigating symptoms such as nasal congestion, runny nose, sneezing, and nasal itching (Fig. 6A–D). Two researchers, Li Yan^[22]^ and Tao Jinghua,^[24]^ reported the consolidated nasal symptom scores, revealing a significant statistical advantage of intranasal acupuncture in improving nasal symptoms, denoted by [MD = −1.59, 95%CI (−1.99, −1.18), *P* < .0001] (Fig. 6E).

### 3.7. Total nonnasal symptom scores (TNNSS)

**Figure 7.**
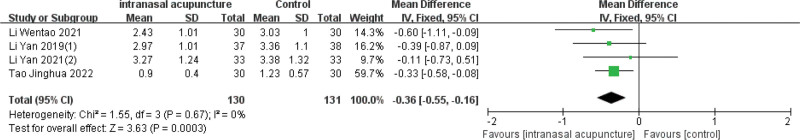
Forest plot of meta-analysis on TNNSS. TNNSS = total nonnasal symptom scores.

The TNNSS score encompasses 5 criteria: nasal discharge from the pharynx, tearing, nasal or eye itching, nasal or oral maxillary discomfort, and headache. The scores for total nonnasal symptoms were reported in 4 studies, comprising 130 children who underwent intranasal acupuncture treatment and 131 in the control group. Since none of the studies provided a breakdown of the TNNSS items, only the aggregate TNNSS score was evaluated. The meta-analysis indicated that intranasal acupuncture demonstrated superiority over other treatments in alleviating total nonnasal symptoms [MD = −0.36, 95%CI (−0.755, −0.16), *P* = .0003]. The findings suggest that intranasal acupuncture is markedly more effective than the control group in reducing scores for total nonnasal symptoms (Fig. 7).

### 3.8. Rhinoconjunctivitis Quality-of-Life Questionnaire (RQLQ) scores

**Figure 8.**
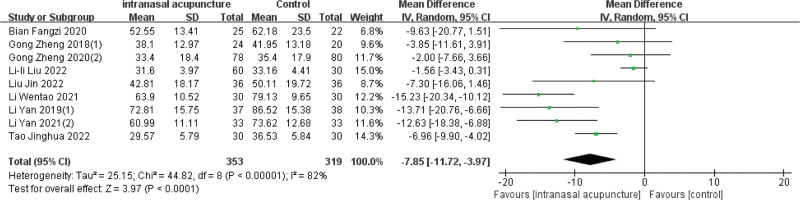
Forest plot of meta-analysis on RQLQ scores. RQLQ = rhinoconjunctivitis quality-of-life questionnaire.

Eight studies assessed RQLQ scores, involving 353 children treated with intranasal acupuncture and 319 in the control group. Data from these trials highlighted a significant enhancement in the quality of life (RQLQ) for the group treated with intranasal acupuncture when compared to the control group [MD = −7.85, 95%CI (−11.72, −3.97), *P* < .001] (Fig. 8).

### 3.9. Recurrence rate

**Figure 9.**
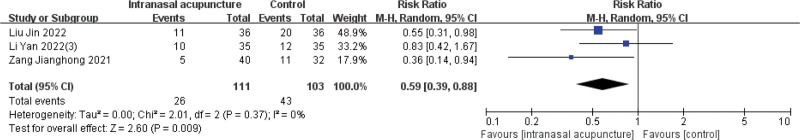
Forest plot of meta-analysis on recurrence rate.

**Figure 10.**
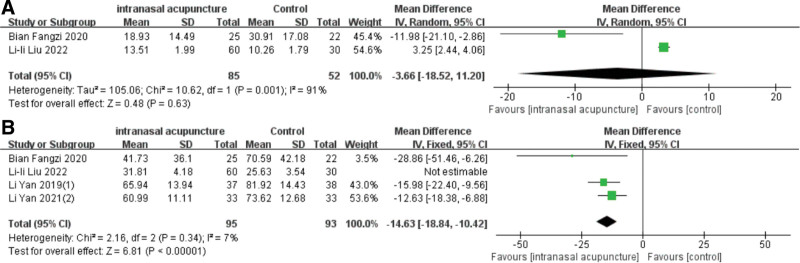
Forest plot of meta-analysis on recurrence rate of VAS scores. VAS = visual analog scale.

**Figure 11.**
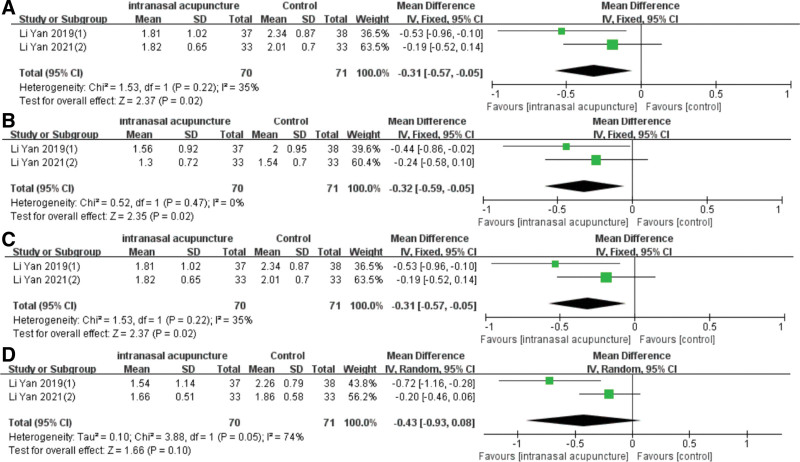
Forest plot of meta-analysis on recurrence rate of TNSS. TNSS = total nasal symptom scores.

A series of 7 studies tracked patients for a duration ranging from 1 to 6 months posttreatment. Three of them documented the number of relapses, which were expressed as dichotomous variables, while 4 incorporated the VAS score, the TNSS or the RQLQ score during follow-up, displayed as continuous variables. The recurrence rates for the intranasal acupuncture and control groups were 23.4% (26/111) and 41.7% (43/103), respectively. The meta-analysis established that the intranasal acupuncture significantly lowered the recurrence rate of AR [RR = 0.59, 95%CI (0.39, 0.88), *P* = .009] (Fig. 9), with a low disparity across trials that reported these outcomes (I2 = 0%, *P* = .37). In the analysis of VAS and RQLQ scores, the random-effects model (I2 = 91%, *P* = .001) (I2 = 96%, *P* < .00001) was utilized to consolidate the effect size. The meta-analysis revealed that 1 month posttreatment, both groups exhibited reduced VAS and RQLQ scores [MD = −3.66, 95%CI (−18.52, 11.2), *P* = .63] [MD = −11.01, 95%CI (−26.01, 4), *P* = .15], however, the difference was not statistically noteworthy (Fig. 10A). Due to only 2 studies reporting VAS scores in the follow-up, it was not possible to analyze the sources of heterogeneity. Excluding the study by Lili Liu resulted in reduced heterogeneity (I2 = 7%, *P* = .34), and the follow-up RQLQ score in the intranasal acupuncture group was significantly lower than that of the control group [MD = −14.63, 95%CI (−18.84, −10.42), *P* < .00001] (Fig. 10B). In total, 141 patients were assessed for TNSS during the follow-up. The data affirmed that intranasal acupuncture notably improved nasal congestion [MD = −0.54, 95%CI (−0.74, −0.34), *P* < .00001], runny nose [MD = −0.32, 95%CI (−0.59, −0.05), *P* = .02], and sneezing [MD = −0.31, 95%CI (−0.57, −0.05), *P* = .02] but not nasal itching (*P* = .10) (Fig. 11A–D).

### 3.10. Adverse reactions

Only 4 trials^[20–22,27]^ documented adverse effects associated with intranasal acupuncture. These trials recorded incidents of nasal bleeding post-acupuncture; specifically, Liu Jin reported 2 cases, and a similar number were noted in Li Yan’s study.^[22]^ The nasal hemorrhage could be halted by pressing on both nasal wings for a duration of 5 minutes. Although Li-Li Liu^[20]^ did not specify the count of nasal bleeding incidents, she highlighted that the volume of blood lost was minimal (<2 mL) and did not compromise the trial. Gong Zheng’s evaluation indicated that the bleeding, primarily of mild to moderate intensity, could be promptly arrested with minor cotton ball caulking lasting 5 minutes. Furthermore, Gong Zheng observed that the pain VAS score within the intranasal acupuncture group was predominantly clustered around 3 points. A minor segment of patients rated the pain above 5 points, suggesting that the majority perceived the pain associated with intranasal acupuncture as mild and bearable.

## 4. Discussion

### 4.1. Summary of main findings

Many studies exploring the potential of acupuncture for AR have been undertaken. However, there hasn’t been a systematic review and meta-analysis examining the efficacy of intranasal acupuncture in treating AR before, to the best of our knowledge. Hence, we hope that this study will pave the way for more effective alternative treatments for AR.

In total, 14 studies were recognized involving 1009 participants, with individual sample sizes spanning between 44 and 158 individuals. The meta-analysis indicated that intranasal acupuncture outperformed nasal sprays, antihistamines, conventional acupuncture protocols, and Traditional Chinese Medicine concoctions in numerous aspects, including in the metrics of VAS, TNSS, TNNSS, and RQLQ scores, along with overall effectiveness. Seven of these studies noted patient relapses, and the meta-analysis demonstrated a substantial decrease in the recurrence rate of AR owing to intranasal acupuncture. Nonetheless, it was observed that intranasal acupuncture had limited long-term effects on alleviating nasal itching in patients afflicted with AR. Moreover, the primary sites chosen for intranasal acupuncture are Neiyingxiang, Biqiu, and Xiabijia, areas abundant with vessels and nerves, which makes pain and bleeding the most prevalent adverse reactions. Yet, based on Gong Zheng’s investigation, it appears that adult patients can generally tolerate these reactions.

AR manifests as a swift allergic response triggered when the body encounters allergens, predominantly characterized by abrupt symptoms such as nasal congestion, runny nose, sneezing, and nasal itching.^[1]^ The focal point of AR treatment should be the prompt alleviation of nasal symptoms.^[34]^ Previous research has established that neither traditional Chinese medicine nor conventional acupuncture delivers quick symptom relief.^[35]^ Although various treatment strategies, like glucocorticoids, antihistamines, immunotherapy, and biological agents have been employed, they come with a host of disadvantages including high costs, fleeting effectiveness, elevated recurrence rates, and numerous side effects. An ancient Chinese medical text,^[36]^ The Yellow Emperor’s Inner Canon, advocates for the strategic selection of acupuncture points based on the disease’s location and the symptomatic nature of the ailment.^[37]^ Contemporary acupuncture treatments largely emphasize remote point selection, neglecting local point selection, leading to suboptimal outcomes. Consequently, some Chinese experts have amalgamated insights from studies on turbinate hypertrophy in AR patients,^[38]^ the traits of parasympathetic stimulation,^[39]^ and the sphenopalatine ganglion acupuncture treatment methodology.^[40]^ This resulted in the proposal of intranasal acupuncture as a viable treatment for AR, a claim substantiated through both clinical and animal experiments.^[20,41]^ Unfortunately, the scant number of studies included in this analysis could not ascertain the most effective acupuncture point or combination for mitigating rhinitis symptoms, limiting our meta-analysis to a generalized evaluation of intranasal acupuncture.

### 4.2. Functions of intranasal acupuncture

Research indicates that the onset of AR is intricately linked to the neuroendocrine-immune network.^[1,42,43]^ Acupuncture has the potential to mitigate nasal symptoms through the reduction of eosinophil infiltration and levels of IgE and inflammatory factors, fostering a balance in Th1/Th2 dynamics, adjusting the nasal mucosal neuropeptide equilibrium, and restraining the activities of the hypothalamic–pituitary–adrenal axis.^[41,44,45]^ Intranasal acupuncture can specifically diminish the nasal mucosa’s Substance P and vasoactive intestinal peptide concentrations, thereby reducing the excitation of sensory and parasympathetic nerves.^[41]^ Furthermore, it can suppress the cyclic-AMP dependent protein kinase A pathway via CD3/CD28 combined stimulation signaling,^[46]^ subsequently elevating IL-2 and interferon γ, reducing IL-4 levels, and curbing Th2 immune responses.^[42,47]^ Additionally, it augments neuropeptide Y content, activating sympathetic nerves, constricting vessels, minimizing gland secretion, and alleviating symptoms like nasal congestion and runny nose.^[48,49]^ Apart from these neuroendocrine-immune network functions, researchers are exploring potential functions of intranasal acupuncture in AR treatment from various angles, including metabolomics and nasal microbiota. For instance, 1 clinical metabolomics study observed the substantial positive impact of intranasal acupuncture on ferrozois, as well as phenylalanine, tyrosine, and tryptophan biosynthesis, compared to oral loratadine syrup.^[50]^ Moreover, a study utilizing 16SrDNA high-throughput technology to inspect nasal secretion microbial flora discovered that intranasal acupuncture could increase the presence of several bacterial groups and modulate species diversity.^[51]^ Consequently, intranasal acupuncture is being proposed as an important acupuncture point for treating AR.

### 4.3. Strengths and limitations

This meta-analysis boasts several merits. Firstly, it stands as the premier systematic review and meta-analysis that comprehensively examines the impact of intranasal acupuncture on nasal symptoms and the quality of life of individuals suffering from AR. Secondly, it accentuates the prolonged effectiveness of intranasal acupuncture, thoroughly assessing the conditions of patients during the follow-up phase. Additionally, aside from Liu Qiaoping work, the majority of the trials were documented in the last 5 years, aligning this research trajectory closely with the current focal points and trends in the field.

Nonetheless, this review is not without its shortcomings. Firstly, the scarcity of high-quality trials is a significant limitation, compounded by a notable deficiency in allocation concealment details in most incorporated trials. The intrinsic differences between intranasal acupuncture and other treatment modalities make it challenging to blind individuals and participants, and it’s equally hard to negate publication bias owing to the limited number of trials included. Secondly, the concentration of the studies in China potentially introduces a regional bias. Thirdly, a considerable disparity was observed in the assessment of the effects of intranasal acupuncture on VAS and RQLQ scores, rendering the outcomes of the random-effects model somewhat unreliable. Fourthly, while the Neiyingxiang, Biqiu, and Xiabijia points are regularly utilized, only the Neiyingxiang point has been extensively investigated in isolation, leaving the efficacy of the Biqiu and Xiabijia points as standalone therapeutic points unexplored. Furthermore, the studies displayed variations in acupuncture frequency and therapy duration, with no subgroup analyses conducted. Consequently, inquiries regarding the most efficacious acupuncture points, frequencies, and intervention durations remain under-analyzed, potentially undermining the validity of our conclusions.

### 4.4. Implications

The repercussions for clinical practice are delineated below. Current guidelines on AR issued by the Japanese Society of Allergology^[3]^ and the European Academy of Allergy and Clinical Immunology^[52]^ endorse acupuncture as a supplementary and alternate treatment avenue for patients exhibiting hormone intolerance or insensitivity. Nevertheless, these clinical directives do not specifically advocate for the utilization of intranasal acupuncture. Considering the favorable attributes of low cost, minimal side effects, user convenience, and the encouraging outcomes of this meta-analysis, we tentatively propose the routine implementation of intranasal acupuncture for AR patients. To enhance its efficacy, the integration of inner incense, nasal mound, and inferior turbinate is recommended.

Looking ahead, the ramifications for subsequent research are elucidated below. Future RCTs should adhere to the Comprehensive Standard for Clinical Trial Reporting (CONSORT) and the Standard for Reporting of Clinical Intervention in Acupuncture (STRICTA) guidelines to enhance the methodological quality and reproducibility of findings. Moreover, the optimal formulation of intranasal acupuncture, along with the ideal frequency and duration of interventions, warrant further investigation. Additionally, future endeavors should encompass high-quality, large-sample, multi-center RCTs to solidify the credibility of intranasal acupuncture as a treatment for AR.

## 5. Conclusion

This analysis indicates that intranasal acupuncture is a potent remedy for AR, for diminishing both nasal and nonnasal symptoms and enhancing patient quality of life. However, these conclusions should be considered with a degree of caution given the limited scope and mediocre quality of the existing literature. Moreover, considering the geographical concentration of the studies in China, it is imperative to facilitate further investigations, encompassing high-quality, large-sample, and multi-center studies in multiple nations to substantiate these preliminary observations.

## Author contributions

**Conceptualization:** Yongjun Li.

**Data curation:** Yongjun Li, Yijie Wang, Yuan Liang.

**Investigation:** Yuan Liang.

**Software:** Yongjun Li.

**Supervision:** Yongjun Li, Xiuying Si.

**Validation:** Yongjun Li.

**Visualization:** Yongjun Li, Yuan Liang, Zhixiang Li, Youpeng Wang.

**Writing – original draft:** Yongjun Li, Yuan Liang.

**Writing – review & editing:** Yongjun Li, Yuan Liang, Youpeng Wang.

## Supplementary Material


