# Supplementary material for: Intranasal acupuncture for allergic rhinitis: A systematic review and meta-analysis
Source: Medicine (Baltimore). 2024 Nov 8;103(45):e40305. doi: 10.1097/MD.0000000000040305 (PMC11557102; doi:10.1097/MD.0000000000040305)
Supplement: Supplementary file 1 [file medi-103-e40305-s001.pdf]

strategies in PubMed.	
ID	Query
#1	" Rhinitis, Allergic "[Mesh] OR " Seasonal allergic rhinitis "[Mesh] OR " Rhinitis, Allergic, Perennial "[Mesh]
#2	(Allergic Rhinitides [Title/Abstract]) OR (Rhinitides, Allergic [Title/Abstract]) OR (Allergic Rhinitis [Title/Abstract]) OR (Allergic Rhinitides, Seasonal [Title/Abstract]) OR (Allergic Rhinitis, Seasonal [Title/Abstract]) OR (Rhinitides, Seasonal Allergic [Title/Abstract]) OR (Rhinitis, Seasonal Allergic [Title/Abstract]) OR (Seasonal Allergic Rhinitides [Title/Abstract]) OR (Pollen Allergy [Title/Abstract]) OR (Allergies, Pollen [Title/Abstract]) OR (Allergy, Pollen [Title/Abstract]) OR (Pollen Allergies [Title/Abstract]) OR (Pollinosis [Title/Abstract]) OR (Pollinoses [Title/Abstract]) OR (Hay Fever [Title/Abstract]) OR (Fever, Hay [Title/Abstract]) OR (Hayfever [Title/Abstract]) OR (Rhinitis, Allergic, Nonseasonal [Title/Abstract])
#3	#1 OR #2
#4	"Intranasal acupuncture"[Mesh]
#5	(Intranasal acupuncture[Title/Abstract])
#6	#4 OR #5
#7	#3 AND #6

National Institute for Health Research

UNIVERSITY of York
Centre for Reviews and Dissemination

Systematic review

*Fields that have an **asterisk (*)** next to them means that they **must be answered. Word limits** are provided for each section. You will be unable to submit the form if the word limits are exceeded for any section. Registrant means the person filling out the form.*

1. * Review title.

Give the title of the review in English

Intranasal acupuncture for allergic rhinitis: A Systematic Review and Meta-Analysis

2. Original language title.

For reviews in languages other than English, give the title in the original language. This will be displayed with the English language title.

3. * Anticipated or actual start date.

Give the date the systematic review started or is expected to start.

18/03/2023

4. * Anticipated completion date.

Give the date by which the review is expected to be completed.

30/04/2023

5. * Stage of review at time of this submission.

This field uses answers to initial screening questions. It cannot be edited until after registration.

Tick the boxes to show which review tasks have been started and which have been completed.

Update this field each time any amendments are made to a published record.

The review has not yet started: Yes

Review stage	Started	Completed
Preliminary searches	No	No
Piloting of the study selection process	No	No
Formal screening of search results against eligibility criteria	No	No
Data extraction	No	No
Risk of bias (quality) assessment	No	No
Data analysis	No	No

Provide any other relevant information about the stage of the review here.

6. * Named contact.

The named contact is the guarantor for the accuracy of the information in the register record. This may be any member of the review team.

Li Yongjun

Email salutation (e.g. "Dr Smith" or "Joanne") for correspondence:

Dr Yongjun

7. * Named contact email.

Give the electronic email address of the named contact.

1531323122@qq.com

8. Named contact address

Give the full institutional/organisational postal address for the named contact.

No. 24 Heping Road, Xiangfang District, Harbin, Heilongjiang Province

9. Named contact phone number.

Give the telephone number for the named contact, including international dialling code.

18804606849

10. * Organisational affiliation of the review.

Full title of the organisational affiliations for this review and website address if available. This field may be completed as 'None' if the review is not affiliated to any organisation.

Heilongjiang University of Traditional Chinese Medicine

Organisation web address:

11. * Review team members and their organisational affiliations.

Give the personal details and the organisational affiliations of each member of the review team. Affiliation refers to groups or organisations to which review team members belong.
NOTE: email and country now MUST be entered for each person, unless you are amending a published record.

Dr Li Yongjun. Heilongjiang University of Traditional Chinese Medicine
Ms Liang Yuan. Heilongjiang University of Chinese Medicine
Mr Li Zhixiang. Heilongjiang University of Chinese Medicine

12. * Funding sources/sponsors.

Details of the individuals, organizations, groups, companies or other legal entities who have funded or sponsored the review.

none

Grant number(s)

State the funder, grant or award number and the date of award

13. * Conflicts of interest.

List actual or perceived conflicts of interest (financial or academic).

None

14. Collaborators.

Give the name and affiliation of any individuals or organisations who are working on the review but who are not listed as review team members. **NOTE: email and country must be completed for each person, unless you are amending a published record.**

15. * Review question.

State the review question(s) clearly and precisely. It may be appropriate to break very broad questions down into a series of related more specific questions. Questions may be framed or refined using PI(E)COS or similar where relevant.

How effective and safe is the intranasal acupuncture for allergic rhinitis compared to traditional treatments

16. * Searches.

State the sources that will be searched (e.g. Medline). Give the search dates, and any restrictions (e.g. language or publication date). Do NOT enter the full search strategy (it may be provided as a link or attachment below.)

Scientific databases (PubMed, Embase, Web of Science, Cochrane Library, CBM, CNKI, Wanfang) were comprehensively searched to identify relevant literature without language restriction.

17. URL to search strategy.

Upload a file with your search strategy, or an example of a search strategy for a specific database, (including the keywords) in pdf or word format. In doing so you are consenting to the file being made publicly accessible. Or provide a URL or link to the strategy. Do NOT provide links to your search **results**.

Alternatively, upload your search strategy to CRD in pdf format. Please note that by doing so you are consenting to the file being made publicly accessible.

Do not make this file publicly available until the review is complete

18. * Condition or domain being studied.

Give a short description of the disease, condition or healthcare domain being studied in your systematic review.

Allergic rhinitis is a non-infectious inflammatory disease of the nasal mucosa mediated by IgE and a variety of immunocompetent cells and factors following exposure to allergens in susceptible individuals.AR can lead to autonomic nervous system disorders, causing nasal mucosal hyperemia and increased gland secretions, with intermittent or persistent nasal congestion, nasal itching, sneezing, and runny nose as the main clinical features. It can be accompanied by itching in the eyes, ears, throat, and even headache, conjunctival hyperemia, loss of smell and other symptoms, which can seriously affect the quality of life of patients. AR is a common clinical chronic nasal disease, affecting 10%~20% of the world's population. With the significant increase in prevalence in recent years, AR has become a major respiratory chronic inflammatory disease, bringing a heavy burden to the social economy.

19. * Participants/population.

Specify the participants or populations being studied in the review. The preferred format includes details of both inclusion and exclusion criteria.

The included population should meet the diagnostic criteria for AR without race and nationality restrictions will be the main participants.

20. * Intervention(s), exposure(s).

Give full and clear descriptions or definitions of the interventions or the exposures to be reviewed. The preferred format includes details of both inclusion and exclusion criteria.

Intranasal acupuncture is a method of treating AR by acupuncture of the Neiyingxiang (EX-HN9), Biqiu and Xiabijia points. The Neiyingxiang point is located at the junction of the nasal mucosa and the bottom of the lower turbinate, and is close to the nasolabial fold. The Biqiu point is located at the forefront of the lateral wall of the middle turbinate in the nasal cavity. Its surface is raised and mound-shaped, and there are adjacent ossified air sacs in it, which are connected to the lacrimal sac. This bulge can be clearly seen through nasal endoscopy. The Xiabijia point consists of the lower turbinate bone and the mucous membrane on its surface, attached to the medial wall of the maxilla and the vertical plate of the palatine bone.

21. * Comparator(s)/control.

Where relevant, give details of the alternatives against which the intervention/exposure will be compared (e.g. another intervention or a non-exposed control group). The preferred format includes details of both inclusion and exclusion criteria.

Conventional treatment, such as nasal spray budesonide, loratadine and Traditional Chinese herbal decoction.

22. * Types of study to be included.

Give details of the study designs (e.g. RCT) that are eligible for inclusion in the review. The preferred format includes both inclusion and exclusion criteria. If there are no restrictions on the types of study, this should be stated.

Randomized controlled trials will be included irrespective of blinding, publication status or language.

23. Context.

Give summary details of the setting or other relevant characteristics, which help define the inclusion or exclusion criteria.

24. * Main outcome(s).

Give the pre-specified main (most important) outcomes of the review, including details of how the outcome is defined and measured and when these measurement are made, if these are part of the review inclusion criteria.

Efficacy of allergic rhinitis was evaluated by using the TNSS scoring method.

Measures of effect

Please specify the effect measure(s) for you main outcome(s) e.g. relative risks, odds ratios, risk difference, and/or 'number needed to treat.

Std. Mean Difference will be the effect measures for main outcomes.

25. * Additional outcome(s).

List the pre-specified additional outcomes of the review, with a similar level of detail to that required for main outcomes. Where there are no additional outcomes please state ‘None’ or ‘Not applicable’ as appropriate to the review

Additional outcomes included VAS scores, TNNSS scores, RQLQ scores, adverse effects and recurrence rate.

Measures of effect

Please specify the effect measure(s) for you additional outcome(s) e.g. relative risks, odds ratios, risk difference, and/or 'number needed to treat.

Std. Mean Difference and relative risks will be the effect measures for main outcomes.

26. * Data extraction (selection and coding).

Describe how studies will be selected for inclusion. State what data will be extracted or obtained. State how this will be done and recorded.

The following data were extracted from each eligible study: first author, country, subjects, number of
participants, sex, age, intervention, comparator, VAS score, TNSS score, TNNSS score, RQLQ score, cure rate,

serum IgE and other related data. To ensure the quality of the meta-analysis, all eligible articles were

independently reviewed and cross-checked="checked" value="1"="checked="checked" value="1"" value="1" by 2 researchers according to standardized

methods. If there was any dissent, another reviewer was consulted. A consensus was reached on the

selection of the final included studies.

27. * Risk of bias (quality) assessment.

State which characteristics of the studies will be assessed and/or any formal risk of bias/quality assessment tools that will be used.

Cochrane risk of bias tool was used to evaluate the risk of bias of each study containing RCT, the following
factors were taken into account: random sequence generation, allocation concealment, blinding of

participants and researchers, blinding of outcome assessments, incomplete outcome data, selective reports

of outcomes and other sources of bias. The risk of bias in each aspect was scored as low, high, or unclear

risk.

28. * Strategy for data synthesis.

Describe the methods you plan to use to synthesise data. This **must not be generic text** but should be **specific to your review** and describe how the proposed approach will be applied to your data. If meta-analysis is planned, describe the models to be used, methods to explore statistical heterogeneity, and software package to be used.

Rev Man 5.4 supplied by the Cochrane was used in this Meta-analysis. The pooled dichotomous data
was expressed as relative risk (RR) and 95% confidence interval (CI). The continuous variables were

expressed as mean standard deviation (MD) and 95% CI Operation. P0.05 was used as the standard to determine the difference to be statistically significant. Q test (P value) and ?² test (I²) were used to determine the heterogeneity. If the test results indicated that there was no significant heterogeneity (P?0.1, I²50%), the fixed-effects model was used for analysis. When there is statistical heterogeneity between studies (P0.1, I²50%), a random effect model is used, and a sensitivity analysis is performed to exclude studies that can cause heterogeneity, and then a meta-analysis is performed. When the included literature 10 A funnel plot was used to test for publication bias.

29. * Analysis of subgroups or subsets.

State any planned investigation of ‘subgroups’. Be clear and specific about which type of study or participant will be included in each group or covariate investigated. State the planned analytic approach.

None

30. * Type and method of review.

Select the type of review, review method and health area from the lists below.

Type of review

Cost effectiveness

No

Diagnostic

No

Epidemiologic

No

Individual patient data (IPD) meta-analysis

No

Intervention

No

Living systematic review

No

Meta-analysis

Yes

Methodology

No

Narrative synthesis

No

Network meta-analysis
No

Pre-clinical
No

Prevention
No

Prognostic
No

Prospective meta-analysis (PMA)
No

Review of reviews
No

Service delivery
No

Synthesis of qualitative studies
No

Systematic review
Yes

Other
No

Health area of the review

Alcohol/substance misuse/abuse
No

Blood and immune system
Yes

Cancer
No

Cardiovascular
No

Care of the elderly
No

Child health
No

Complementary therapies

No

COVID-19
No

Crime and justice
No

Dental
No

Digestive system
No

Ear, nose and throat
Yes

Education
No

Endocrine and metabolic disorders
No

Eye disorders
No

General interest
No

Genetics
No

Health inequalities/health equity
No

Infections and infestations
No

International development
No

Mental health and behavioural conditions
No

Musculoskeletal
No

Neurological
No

Nursing
No

Obstetrics and gynaecology

No

Oral health
No

Palliative care
No

Perioperative care
No

Physiotherapy
No

Pregnancy and childbirth
No

Public health (including social determinants of health)
No

Rehabilitation
No

Respiratory disorders
No

Service delivery
No

Skin disorders
No

Social care
No

Surgery
No

Tropical Medicine
No

Urological
No

Wounds, injuries and accidents
No

Violence and abuse
No

31. Language.

Select each language individually to add it to the list below, use the bin icon to remove any added in error.

English

There is not an English language summary

32. * Country.

Select the country in which the review is being carried out. For multi-national collaborations select all the countries involved.

China

33. Other registration details.

Name any other organisation where the systematic review title or protocol is registered (e.g. Campbell, or The Joanna Briggs Institute) together with any unique identification number assigned by them. If extracted data will be stored and made available through a repository such as the Systematic Review Data Repository (SRDR), details and a link should be included here. If none, leave blank.

34. Reference and/or URL for published protocol.

If the protocol for this review is published provide details (authors, title and journal details, preferably in Vancouver format)

Add web link to the published protocol.

Or, upload your published protocol here in pdf format. Note that the upload will be publicly accessible.

No I do not make this file publicly available until the review is complete

Please note that the information required in the PROSPERO registration form must be completed in full even if access to a protocol is given.

35. Dissemination plans.

Do you intend to publish the review on completion?

No

Give brief details of plans for communicating review findings.?

36. Keywords.

Give words or phrases that best describe the review. Separate keywords with a semicolon or new line. Keywords help PROSPERO users find your review (keywords do not appear in the public record but are included in searches). Be as specific and precise as possible. Avoid acronyms and abbreviations unless these are in wide use.

37. Details of any existing review of the same topic by the same authors.

If you are registering an update of an existing review give details of the earlier versions and include a full bibliographic reference, if available.

38. * Current review status.

Update review status when the review is completed and when it is published.New registrations must be ongoing so this field is not editable for initial submission.

Please provide anticipated publication date

Review_Ongoing

39. Any additional information.

Provide any other information relevant to the registration of this review.

40. Details of final report/publication(s) or preprints if available.

Leave empty until publication details are available OR you have a link to a preprint (NOTE: this field is not editable for initial submission). List authors, title and journal details preferably in Vancouver format.

Give the link to the published review or preprint.
